# Robust Moth-Inspired Algorithm for Odor Source Localization Using Multimodal Information

**DOI:** 10.3390/s23031475

**Published:** 2023-01-28

**Authors:** Shunsuke Shigaki, Mayu Yamada, Daisuke Kurabayashi, Koh Hosoda

**Affiliations:** 1 Graduate School of Engineering Science, Osaka University, 1-2 Machikaneyama-cho, Toyonaka-ku, Osaka 560-0043, Japan; 2 Department of Systems and Control Engineering, Tokyo Institute of Technology, 2-12-1 Ookayama, Meguro-ku, Tokyo 152-8552, Japan

**Keywords:** odor-source localization, insect VR system, multisensory motor integration, moth-inspired algorithm

## Abstract

Odor-source localization, by which one finds the source of an odor by detecting the odor itself, is an important ability to possess in order to search for leaking gases, explosives, and disaster survivors. Although many animals possess this ability, research on implementing olfaction in robotics is still developing. We developed a novel algorithm that enables a robot to localize an odor source indoors and outdoors by taking inspiration from the adult male silk moth, which we used as the target organism. We measured the female-localization behavior of the silk moth by using a virtual reality (VR) system to obtain the relationship between multiple sensory stimuli and behavior during the localization behavior. The results showed that there were two types of search active and inactive depending on the direction of odor and wind detection. In an active search, the silk moth moved faster as the odor-detection frequency increased, whereas in the inactive search, they always moved slower under all odor-detection frequencies. This phenomenon was constructed as a robust moth-inspired (RMI) algorithm and implemented on a ground-running robot. Experiments on odor-source localization in three environments with different degrees of environmental complexity showed that the RMI algorithm has the best localization performance among conventional moth-inspired algorithms. Analysis of the trajectories showed that the robot could move smoothly through the odor plume even when the environment became more complex. This indicates that switching and modulating behavior based on the direction of odor and wind detection contributes to the adaptability and robustness of odor-source localization.

## 1. Introduction

Using an odor to identify the object at its source is important because doing so can help authorities find drugs and explosives in hidden places; it can also be applied to rescue human lives at disaster sites. Currently, dogs play that role, but training them to be scent-detection dogs or scent hounds [1] takes a long time and has a high cost. In addition, because dogs are living creatures, they cannot enter dangerous areas, and their sense of smell deteriorates due to to fatigue and aging. Therefore, developing a robot system that can replace dogs is an urgent issue. Because most animals, including dogs, use the chemical properties of their surroundings to survive and thrive, they are adept at using odors [2]. However, odors do not propagate by wave motion but by diffusion of chemicals into space via air, which has the characteristic of losing orientation and an inability to create an orderly gradient. Therefore, it is still difficult for a robot to find an odor source by relying on information about the odor, and robot olfaction has not been established. To address the odor-source search problem from an engineering viewpoint, methods have been proposed that use statistical properties to estimate the odor source or allow the robot to mimic the odor-source search behavior of animals [3,4]. Using statistical properties makes running these methods in real time difficult due to high computational costs, and there is a risk of performance degradation in situations in which the environment changes significantly. In contrast, the method to inspire animal behavior can run in real time because it has relatively low computational costs. However, because it merely inspires behavior in response to odor stimuli, it is yet to achieve a level of localization performance comparable to that of animals. To localize to the odor source with high accuracy in real time, it is important to appropriately perceive complex changes from moment to moment and appropriately select actions according to the situation.

In recent years, methods of investigating the adaptability or robustness of animals during localization or navigation behavior by using virtual reality (VR) have gained traction [5]. Experiments using VR have proven to be effective for mammals and insects [6,7,8]. The observation of insects by using virtual reality has revealed multisensory motor-integration mechanisms during navigation. It has been clarified that an insect moves efficiently by using odor and multiple pieces of sensory information in localization behavior to an odor source [8]. This is because the insect modulates its behavior according to the input timing and amount of multiple sensory stimuli, which may be the key to adaptive behavior in complex environments. Although there have been studies that have shown that multiple sensory information (odor and wind) can be used to search odor sources, the scope of application is limited because it is a simple algorithm that reflexively switches actions according to the detection status of multiple sensors [9,10].

Therefore, we investigated multiple sensory-motor integrations of an actual insect, clarified what kind of behavioral modulation is performed during odor-source localization behavior, and reconstructed it as an algorithm. Moreover, we implemented the algorithm on a mobile robot and evaluated localization performance when the complexity of the environment changed stepwise. Thus, we experimentally verified the influence of the behavioral modulation mechanism based on multisensory information inherent in the insect on the function of odor-source localization. We employ bioinspired algorithms proposed in previous studies for comparison. Most of the conventional bioinspired algorithms focused on the timing of switching of behavioral states, and their effectiveness has been demonstrated in a wind tunnel environment. To make practical use of bioinspired algorithms, it is important that they work well in complex environments, and this requires that the algorithms approach the original performance of an insect. For that purpose, it is essential to incorporate a behavior-modulation mechanism, according to the situation, rather than simply switching behavior into the model. This study provides an example of a research framework for measuring and modeling the adaptive behavior of an insect, which contributes to the future development of the bioinspired robotics field.

## 2. Problem Statement

This study models the odor-source localization behavior of an insect that uses multimodal information and verifies the effectiveness of the proposed model by a constructive approach. We focused on the female-localization behavior of an adult male silk moth, *bombyx mori* (hereafter referred to simply as the “silk moth”). The silk moth elicits exploratory behavior when it detects the sex pheromone bombykol [11], and can localize to a female with high accuracy by intermittently obtaining odor information [12]. It has been reported that this female localization behavior is modulated by the odor frequencies in space and other sensory stimuli [8,12,13]. This behavior-modulation mechanism should play a role in high search performance. However, whether it can function correctly when reconfigured as an artificial system such as a robot remains unclear.

Therefore, we first investigate how multimodal information during the female-localization behavior of a silk moth influences behavioral modulation. Specifically, the relationship between vision and wind information, in addition to odor and behavioral modulation, is measured by using a VR system. For the results of VR experiments, we analyzed and algorithmized the relationship between multisensory stimulus input and behavioral output. We implemented the proposed algorithm on a ground-running robot, conducted odor-source localization experiments in indoor and outdoor environments, and evaluated the algorithm’s performance. As comparison models, moth-inspired algorithms [9,14] that have been proposed in previous studies are used, and we investigated whether localization performance is improved compared to these algorithms. One of the compared algorithms is the surge-zigzagging algorithm inspired by the search behavior of the silk moth, which switches between translation and rotational behavior based on odor detection. The second is the surge-cast algorithm inspired by the search behavior of a flying insect (e.g., the hawkmoth), which demonstrates the importance of moving upwind during odor detection. These reactive algorithms are known to work well in a laminar environment such as a wind tunnel, but it is questionable whether they can maintain their performance in an outdoor environment. In more complex environments, it is likely that more behavioral modulation than simple switching of behavioral states is required. In this study, we conduct odor-source localization experiments in complex environments, including the outdoors, to demonstrate the effectiveness of an algorithm that describes insect behavior in more detail, by using odor-source localization, the localization time, and the meandering degree of the trajectory as evaluation indices.

## 3. Biological Experiment for Odor-Source Localization Behavior Analysis

### 3.1. Study Insect

We employed male silk moths, *Bombyx mori*, in their adult form. The body length of the silkworm moth was approximately 25 mm, and it had two antennae on its head for detecting the sex pheromone bombykol [11]. The male moth reaches the female moth by walking because it is a flightless insect [12].

Silk moths (*Bombyx mori*; Lepidoptera: Bombycidae) were purchased from Ehine Sansyu Co., Japan. Adult male moths were cooled to 16 °C one day after eclosion to reduce their activity and were tested within 2–7 d of eclosion. Before the experiments, the moths were kept at room temperature (25–28 °C) for at least 10 min. All the experiments were conducted from 9 am to 5 pm because moths are diurnal insects, and the serotonin level in the brain is highest around these times [15].

### 3.2. Experimental Setup and Design

**Figure 1:**
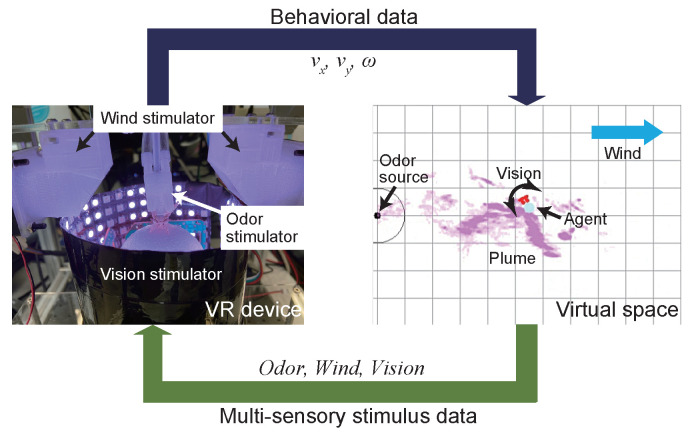
Schematic diagram of VR system for an insect. The VR device has an odor, wind, and visual stimulators. A VR device was connected to a virtual space on the computer, allowing the insect to perform odor-source localization virtually.

Experiments to measure behavioral changes in response to multiple sensory stimuli by using a silk moth were conducted by using an insect VR system as shown in Figure 1 [8]. The insect VR system is a behavior-measurement device based on a tethered measurement device [16]. It can provide stimuli such as odor, vision, and wind direction simultaneously and continuously to the silk moth. Moreover, the silk moth can perform female-localization behavior virtually because the insect VR system is connected to a virtual space built into a computer. Odor diffusion in the virtual space can be reproduced more realistically by recording the diffusion of odor (smoke) in advance by using particle image velocimetry. The environmental information detected by the agent (virtual silk moth) in the virtual space was immediately presented to the real silk moth because the virtual space and the behavior measurement device were linked. For the odor stimulus, an odor outlet was installed above each antenna of the silk moth. It provided odor stimuli independently to the left and right antennae. Moreover, the silk moth detected the flow of scenery with its compound eyes; therefore, an LED array that can provide an optical flow was placed around the silk moth, and the direction of the optical flow provided to the silk moth was controlled by the moving direction of the agent. Furthermore, the direction of the wind stimulus was set to be provided from the front, back, left, and right of the silk moth. The posture of the agent in the virtual space determines the presentation direction. A hawkmoth is a flying insect that moves approximately 0.7 m/s during odor-source localization [17], and it is possible that it detects a wind speed of over 0.7 m/s. Moreover, it has been reported that the odor-source localization behavior of the silk moth [18], was affected when a wind speed of 1.0 m/s was provided; therefore, the wind speed of the stimulation was set to 1.0 m/s. The control of each stimulator and the trajectory recording are performed at 30 Hz.

By using the VR system, we conducted behavioral experiments under three stimulus conditions shown in Table 1. In addition, plus in Table 1 indicates that the stimulus was provided to the silk moth from the same direction as the sensory stimulus detected by the agent. However, the minus signs in Table 1 indicate that the stimulus was presented to the silk moth from the opposite direction of the agent’s detection direction. For example, if the agent detects the wind from the front in the virtual space, we provide the wind stimulus to the silk moth from the back. In addition to these conditions, the VR experiment was carried out under the condition that the agent starts from a point 300 mm away from the position of the odor source in the virtual space, with the initial posture tilted by 30°.

### 3.3. Extraction of Behavioral Modulation

**Figure 2:**
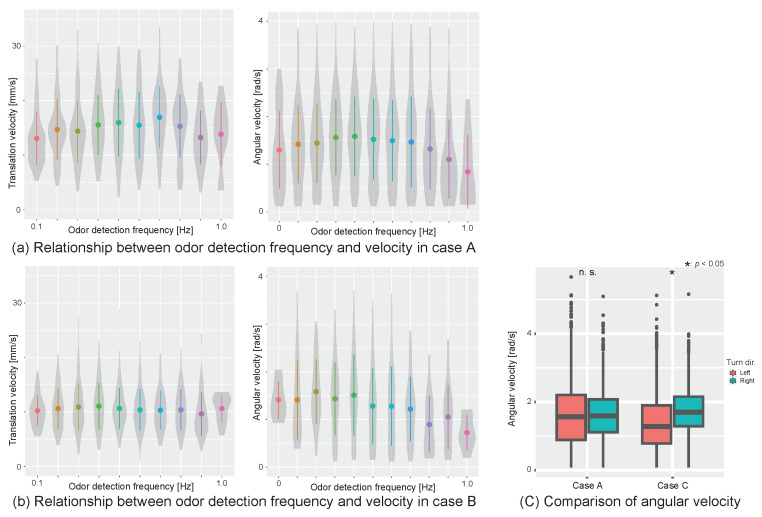
Analysis of a silk moth velocity change in VR experiments. (**a**) Odor detection frequency and changes in translation and angular velocity in Case A. (**b**) Odor detection frequency and changes in translation and angular velocity in Case B. (**c**) Effect of angular velocity on visual stimulus change.

Figure 2 shows the change in velocity when the experiment was conducted under the three conditions in Table 1. The horizontal axis of Figure 2a,b are the odor frequency (Hz), which indicates how often the agent detects the odor per unit of time. The odor frequency was adopted because it has been reported in previous research that an insect’s behavior changes according to the frequency at which the odor is detected [19]. When the translational speed is set on the vertical axis, we found that if the odor and wind detection directions are the same, the search is actively performed at up to 0.7 Hz, and the speed decreases to over 0.7 Hz (Figure 2a). However, when the odor and wind detection directions were different, the velocity was always low regardless of the odor frequency (Figure 2b). The graph of angular velocity on the vertical axis (Figure 2a,b) shows that the velocity peaked at 0.4 Hz when the wind and odor were detected in the same direction but peaked at 0.2 Hz when the wind and odor were detected in different directions. In an experiment with visual stimuli operation, the left–right angular velocity balance was maintained when the visual stimuli were given correctly, but when the visual stimuli were different from the behavioral output, the left–right angular velocity balance was disrupted (Figure 2c). This may be because the silk moth controls postural through vision.

This suggests that the silk moth localizes to the female by switching between two modes during localization behavior. One is an active search mode, and the other is an inactive search mode. From the results of biological experiments, we found that these modes are switched depending on the detection direction of the odor and the wind. When the detection direction of the odor and the wind match, it has reached active search mode and actively tries to approach the female by increasing the velocity depending on the odor detection frequency. However, if the odor and wind direction do not match, it is more likely to move out of the odor plume or move in the wrong direction; therefore, it transits the inactive search mode, slowly and deliberately examining information about the environment at a low speed. In the next chapter, we experimentally verified the effect of this silk moth’s search-mode switching mechanism on the robot localization performance.

## 4. Algorithm Performance Verification Experiment

### 4.1. Robust Moth-Inspired Algorithm

**Figure 3:**
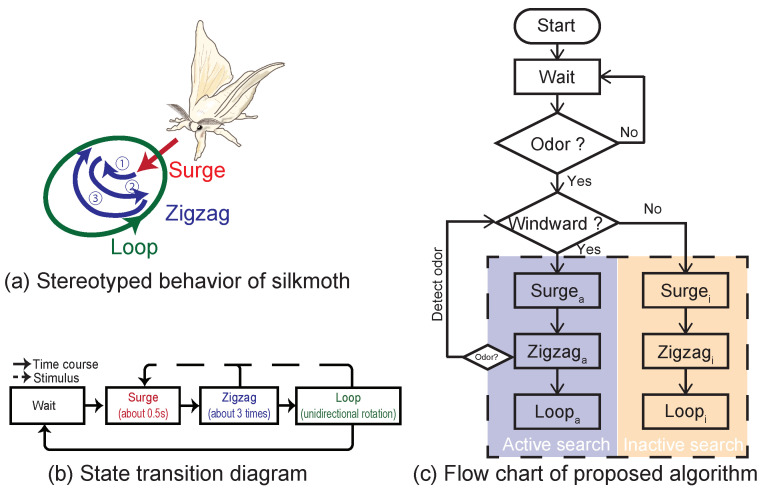
Outline of the typical behavioral patterns during female localization of the silk moth and flowchart of the proposed algorithm.

This section explains a proposed algorithm. Previously, Kanzaki et al. [12] reported that the localization behavior of a silk moth comprises a stereotyped behavioral pattern. Based on this report, a surge-zigzagging algorithm [12,14] was proposed for odor plume tracking, and our proposed algorithm here is also based on this algorithm. As shown in Figure 3a,b, the surge-zigzagging algorithm comprises three states: surge, zigzag, and loop. Surge is a transitional state upon detecting an odor stimulus and moving in the detected direction. The surge duration is approximately 0.5 s [12], and after 0.5 s, it transitions to the zigzag state, which turns left and right. In the zigzag state, it turns left and right three times on average and transitions to a loop state in which it continues to rotate in a certain direction on the fourth turn. In either surge, zigzag, or loop, when an odor stimulus is detected, it transits to the surge state. In the conventional surge-zigzagging algorithm, it takes a constant velocity in any behavioral state.

In the algorithm proposed here, the basic configuration of each behavioral state (surge, zigzag, loop) is the same. However, the velocity of each state is modulated according to the detection status of the odor and the wind sensor. In other words, an “active search mode” is selected when the odor sensor receives wind from the front when it detects an odor, and an “inactive search mode” is selected when the odor sensor detects wind from a direction other than the front when it detects the odor (Figure 3c). The velocity of each mode is modulated in the same way as that of the silk moth in the VR experiment. However, considering the lowest speed of the robot, the minimum and maximum speeds were determined. Velocity modulation in each mode is as follows (Equation (1)):

(1) $$\begin{array}{cc} K_{v/\omega }\left(f\right) & =a_{v/\omega }\times f+b_{v/\omega }. \end{array}$$

Each Equation (1) parameter is set to Table 2. This equation was derived by fitting the actual silk moth behavior experiment data, and each parameter was empirically adjusted according to the characteristics of the robot. This algorithm is called the robust moth-inspired (RMI) algorithm for convenience.

The RMI algorithm is compared with the surge-zigzagging (SZL) and surge-cast (SC) algorithms proposed in previous studies [9,14]. The behavior rule of SZL is almost the same as that of RMI, but it always moves at a constant velocity regardless of the situation. The straight velocity of SZL was set to 150 mm/s and the rotational velocity to 1.5 rad/s. The SC algorithm, another comparison model, mimics the odor-source localization behavior of flying insects. It comprises behavior that moves upwind (surge) when it detects an odor and moves crosswind (cast) when it loses the odor to search for the next piece of odor information. The straight velocity of the SC algorithm was set to 150 mm/s, and its angular velocity was set to 1.5 rad/s.

These three algorithms are implemented in an autonomous robot, and the next section describes detailed information on the robot.

### 4.2. Mobile Robot Configuration

**Figure 4:**
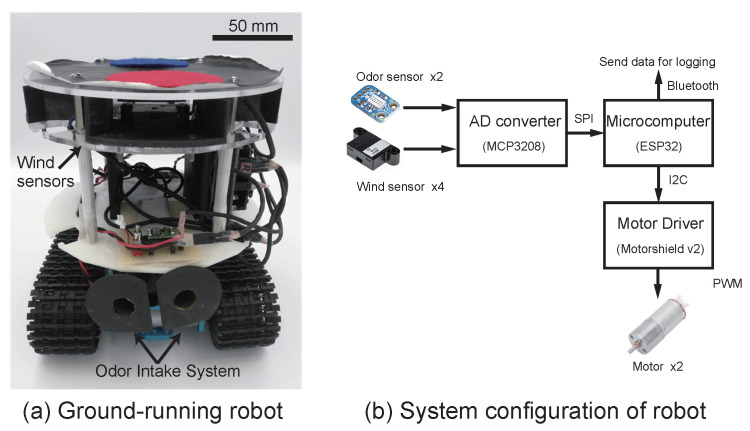
Image of the constructed robot and the system configuration.

Here, a robot with a crawler-type drive system was constructed to move even when the ground is uneven because the robot is expected to conduct experiments indoors and outdoors (Figure 4a). The robot has two alcohol sensors (MICS-5524, Amphenol SGX Sensortech, Switzerland) and four wind sensors (D6F-W01A1, Omron, Japan). The silk moth actively takes in odor in its direction by flapping its wings, which has been reported to be effective in localization behavior toward the female [20,21]. Thus, we did not simply mount the alcohol sensors on the robot but installed them into an active odor-intaking system [14]. Because the odor sensors have a low temporal resolution problem, we addressed this issue by using an inverse model-estimation method based on the ARX model [22]. The directionality of the wind sensors was increased by installing a partition between each wind sensor [18,23], allowing them to discriminate between four different directions.

The processor is an ESP32 (Espressif Systems, China) capable of Bluetooth communication, and the data logs from the sensors are sent to a personal computer (Figure 4b). By implementing algorithms in the ESP32, the robot can autonomously localize the odor source. The sampling period of each sensor is set to 10 ms.

### 4.3. Experiment Field Condition

To investigate the effectiveness of the proposed algorithm, we conducted odor-source localization experiments in the following three environmental scenarios.

**Figure 5:**
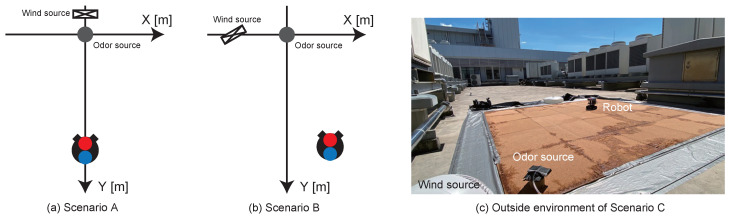
Schematic diagram of the experimental field for each scenario.

1. Scenario A: Wind is generated from the same direction as the odor source in an indoor environment (Figure 5a).
2. Scenario B: The fan generates airflow from a different direction from the odor source in an indoor environment (Figure 5b).
3. Scenario C: Experiment in an outdoor environment (Figure 5c).

Scenario A is the simplest environment, whereas Scenario B is more complex than Scenario A because the odor and the wind source are set to different positions. Scenario C was conducted on the roof of a building, where the wind blows strongly; therefore, scenario C may occur with unpredictable odor dynamics. We evaluated the robustness of the proposed algorithm in these three environments based on the search success rate, search time, and trajectory analysis. In all scenarios, the odor source was placed at the origin (*x*, *y*) = (0, 0) [m]. Ethanol was used as the odor source from the viewpoint of its safety and availability. The ethanol was placed in a gas wash bottle, and the air was supplied at a very low flow rate of 1.0 L/min to generate the odor. In Scenarios A and C, the wind source was placed at (*x*, *y*) = (−0.5, 0) [m], and in Scenario B, the wind generator was placed at (*x*, *y*) = (0, −0.3) [m]. The wind source was a fan (YLS-18, Yamazen, Osaka, Japan) that generates wind speeds of approximately 1.0 m/s. In Scenario A, the robot was placed at about (*x*, *y*) = (1.5, 0) [m]. In Scenarios B and C, the robot started its search at about (*x*, *y*) = (1.5, 0.3) [m]. Localization was considered successful when the robot entered within a radius of 0.1 m of the odor source and failed when the robot left the field. Moreover, a time limit of 3 min was set, and the robot was considered to have failed in localization if it exceeded the time limit. The trajectory of the robot was recorded by using a video camera. The experiment was repeated 20 times for each scenario and algorithm.

### 4.4. Experiment Results

**Figure 6:**
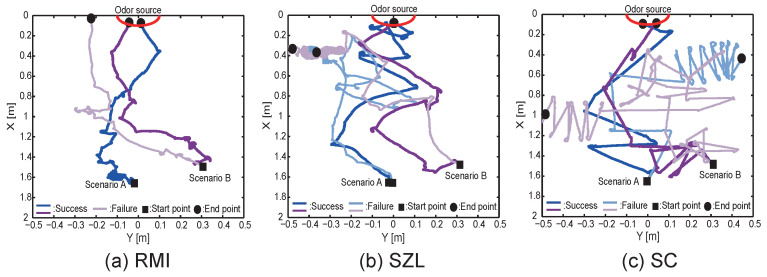
Typical trajectories for scenarios A and B.

**Figure 7:**
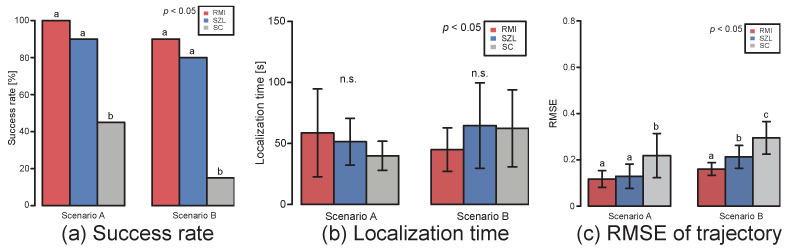
Results of 20 repeated experiments. Results of (**a**) search success rate, (**b**) localization time, and (**c**) RMSE for trajectory. Groups sharing the same letter are not significantly different.

First, we show the results for Scenarios A and B, which are indoor environments. The typical trajectory of each algorithm is shown in Figure 6. Figure 6a–c shows the trajectories of RMI, SZL, and SC, respectively. The red line in the figure represents the location of the odor source, and the blue and purple lines represent Scenarios A and B, respectively. The pale-colored lines indicate the trajectories of failure searches. According to the RMI trajectory, we found that the robot moves smoothly within the odor plume even if the wind source is set at a different position from the odor source. In contrast, the SZL, which uses only odors, moves greatly in the y-axis direction while approaching the odor source. The robot could go out of the odor plume owing to the large deviation, and localization could fail. The SC algorithm that moves in the upwind direction owing to odor detection was found to smoothly localize to the odor source when the upwind detection was successful. However, when it falls into a situation in which the windward detection is unsuccessful, the robot often moves in a different direction. Figure 7a,b show the search success rate and search time for Scenarios A and B when localization experiments were performed 20 times. The horizontal and vertical axes of Figure 7a indicate each scenario and the success rate, respectively. Moreover, the vertical axes of Figure 7b indicate the localization time. Red, blue, and gray bars represent the RMI, SZL, and SC algorithms, respectively. The RMI algorithm had a superior success rate under all scenarios (Fisher’s exact test, *p* < 0.05). However, the SC algorithm tended to have a lower search success rate in all scenarios. In terms of localization time (Steel–Dwass test, *p* < 0.05), when the odor source and the wind source were facing the same direction (Scenario A), the SC algorithm, with its strategy of moving upwind, was found to reach the odor source the fastest. However, when the odor and wind source were located in different locations (Scenario B), there was little difference between the RMI and SC algorithms. Focusing on the SZL algorithm, we found that it reached the odor source faster than the RMI in Scenario A. However, it tended to have the longest localization time in environments in which the odor was diffused in different directions. Figure 7c shows the results of calculating the root mean squared error (RMSE) for the trajectory. The following equation represents the calculation of the RMSE,

(2) $$\mathrm{RMSE}=\sqrt{\frac{1}{n}\sum_{i=0}^{n-1}{\left(y_{i}-y_{\mathrm{odor}}\right)}^{2}},$$

where n, $y_{i}$, and $y_{\mathrm{odor}}$ are the total number of steps, the y-coordinate of the robot at time i, and the y-coordinate of the odor source, respectively. This allows quantitative expression of how much meandering in the crosswind direction was involved in the localization. The smaller the value of RMSE, the more smoothly it moved through the odor plume. Because the RMSE value of the RMI algorithm is significantly small in Scenarios A and B (Steel–Dwass test, *p* < 0.05), the RMI algorithm can search without depending on the location relationship of the odor and the wind source.

**Figure 8:**
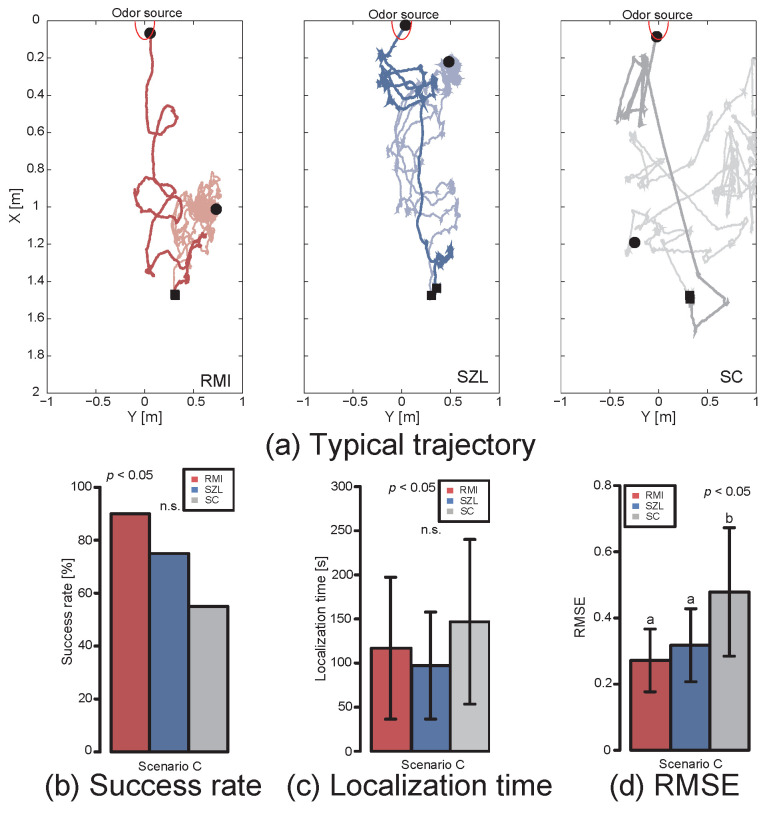
Experimental results for Scenario C. (**a**) Typical trajectory for each algorithm. (**b**) Search success rate. (**c**) Localization time. (**d**) RMSE of trajectory. Groups sharing the same letter are not significantly different.

Next, we show the experimental results for Scenario C in the outdoor environment. Figure 8a–d shows the trajectory, search success rate, localization time, and RMSE of the trajectory, respectively. In the outdoor experiment, as in the indoor experiment, RMI had the highest search success rate, and SC had the lowest (Fisher’s exact test, *p* < 0.05). By contrast, there was almost no difference in the localization time (Steel–Dwass test, *p* < 0.05). However, we found that the RMI, which modulates its velocity according to the odor and wind conditions, moved more smoothly in terms of the RMSE of the trajectory (Figure 8d, Steel–Dwass test, *p* < 0.05).

These results indicate that modulating the velocity of movement according to the direction of odor and wind detection and the odor-detection frequency is effective in indoor and outdoor environments. According to the success rate and RMSE of SC that moves in the upwind direction, it is likely that accurately obtaining wind direction with a mobile robot is as difficult as detecting the odor. Thus, the results suggest that an algorithm that utilizes wind information dominantly to move precisely may be disadvantageous in complex environments. In other words, in a well-organized environment such as Scenario A or a wind tunnel, SZL and SC are also effective. However, in situations in which the direction of odor and wind do not coincide owing to turbulence, it is suggested that it is important to modulate behavior according to the situation.

## 5. Conclusions

We investigated the relationship between multiple sensory stimuli and behavioral changes in a silk moth during female-localization behavior. We proposed a novel moth-inspired algorithm that can run indoors and outdoors. The behavior of the silk moth during female localization was measured by using an insect VR system, and changes in behavior were analyzed when the silk moth was exposed to different odor, visual, and wind stimuli conditions. The results showed that the silk moth compared the direction of odor and wind detection, actively searched when the directions were the same, and inactively searched when the directions were different. We constructed an algorithm incorporating this switching of search strategy depending on the sensing situation, implemented it on a ground-running robot, and conducted odor-source localization experiments. The algorithm’s effectiveness was confirmed by changing the positions of the odor and the wind source in the robot experiments. Experiments were conducted in an outdoor environment in which airflow could not be controlled, and the algorithm’s robustness was evaluated. The results showed that the localization performance of the proposed algorithm was the best in all environments. The RMSE was calculated to determine how far the trajectory deviated from the odor source line, and we found that the proposed algorithm had the lowest value in all environments. This indicates that the proposed algorithm is trying to keep moving within the odor plume even in different environments. This effect is considered to have contributed to the improvement in localization performance. The complexity of the odor is expected to change depending on the environmental conditions; therefore, to deal with this, we need to properly understand the situation with multiple sensors, appropriately switch search strategies, and modulate velocity according to the situation. Here, by switching and modulating behavior based on the difference between the direction of odor and wind detection, we demonstrated that the robot performed high localization performance even when the wind blew from a direction other than that of the odor source.

In the current environment, we set the odor and wind source in different locations, and the experiment was conducted in an open field with no obstacles. Therefore, we could not examine whether the proposed algorithm can handle air turbulence caused by obstacles. In the future, we plan to investigate odor source localization in environments with scattered obstacles and larger spaces. In addition, we will investigate whether the system can be applied to spaces where multiple odor sources exist in the future.

## Figures and Tables

**Table 1:** Conditions for VR experiment. The “+” indicates that the detection direction of the agent and the direction of stimulus presentation to the silk moth is the same. The “−” indicates that the detection direction of the agent and the direction of stimulus presentation to the silk moth are exactly opposite.

	Stimulation	Odor	Wind	Vision
Case				
A		+	+	+
B		+	−	+
C		+	+	−

**Table 2:** Each parameter list of Equation (1). The left side is the parameter for active search, and the right side is the parameter for inactive search.

	Active Mode	$a_{v}$	$a_{\omega }$	$b_{v}$	$b_{\omega }$		Inactive Mode	$a_{v}$	$a_{\omega }$	$b_{v}$	$b_{\omega }$
*f* [Hz]						*f* [Hz]					
≤0.7		53.6	0.888	129	1.82	≤0.7		0.0	1.13	105	1.85
>0.7		−114	246	−1.59	2.81	>0.7		0.0	−1.30	105	2.34

## Abbreviations

The following abbreviations are used in this manuscript:

VR	Virtual Reality
RMI	Robust Moth-Inspired
SZL	Surge-zigzag-loop
SC	Surge-Cast
RMSE	Root Mean Squared Error
